# The complete mitochondrial genome of the tropical sea urchin *Tripneustes gratilla* (Linnaeus, 1758) with two spine colors

**DOI:** 10.1080/23802359.2023.2298091

**Published:** 2024-01-03

**Authors:** Heng Wang, Xiying Zhang, Jiahui Yao, Debao Gang, Yuting Zeng, Mingguang Mao, Jielan Jiang

**Affiliations:** a Key Laboratory of Mariculture & Stock Enhancement in North China’s Sea, Ministry of Agriculture and Rural Affairs, Dalian Ocean University, Dalian, China; b Yazhou Bay Innovation Institute, Hainan Tropical Ocean University, Sanya, China

**Keywords:** Mitochondrial genome, sea urchin, *Tripneustes gratilla*, spine color variation

## Abstract

The complete mitochondrial genomes of two spine-color individuals, red and white, of the tropical sea urchin species *Tripneustes gratilla* (Linnaeus, 1758) were sequenced on Illumina system platform. The red-spined species had a genome size of 15,774 bp, while the white-spined species had a genome size of 15,723 bp. Both genomes contained 13 protein-coding genes, 2 rRNA genes, and 22 tRNA genes. The GC composition in both species was above 40%. In order to investigate the phylogenetic relationships of two different spine-color individuals, a comprehensive analysis was conducted using eight complete mitochondrial genomic sequences of the genus Tripneustes on the software MEGA X. It was observed that the two spine color types of *T. gratilla* species showed a high similarity of 98.91%. However, different color-spined species of *T. gratilla* were found in separate branches of the phylogenetic tree of the same sea urchin species.

## Introduction

*Tripneustes gratilla* is a tropical sea urchin species commonly known as the Collector Urchin. It belongs to the family Toxopneustidae and is found at depths of 0 to 75 meters in the waters of the Indo-Pacific region, from east Africa to southeast Asia, north to South Korea, Japan and Hawaii, south to Australia and New Zealand, to as far east as Galapagos Islands (Lane et al. 2001; Schoppe 2000; Kroh and Mooi 2023). This species are dark in color, usually bluish-purple with white spines, and the pedicles are also white, with a dark or black base. The spines of some specimens are wholly orange, while those of others are only orange-tipped or completely white, which is associated with adaptability to the environment (Clark 1921; Toha et al. 2015). The presence of two distinct color types, red-spined and white-spined, in *T. gratilla* is a common occurrence in Hainan Province, China. Due to a lack of comprehensive genomic data, the evolutionary history, taxonomical relationships, and genetic distinctions of *T. gratilla* with different colorations have remained unclear, impeding our understanding of their evolutionary trajectories.

## Materials and methods

**Figure 1.**
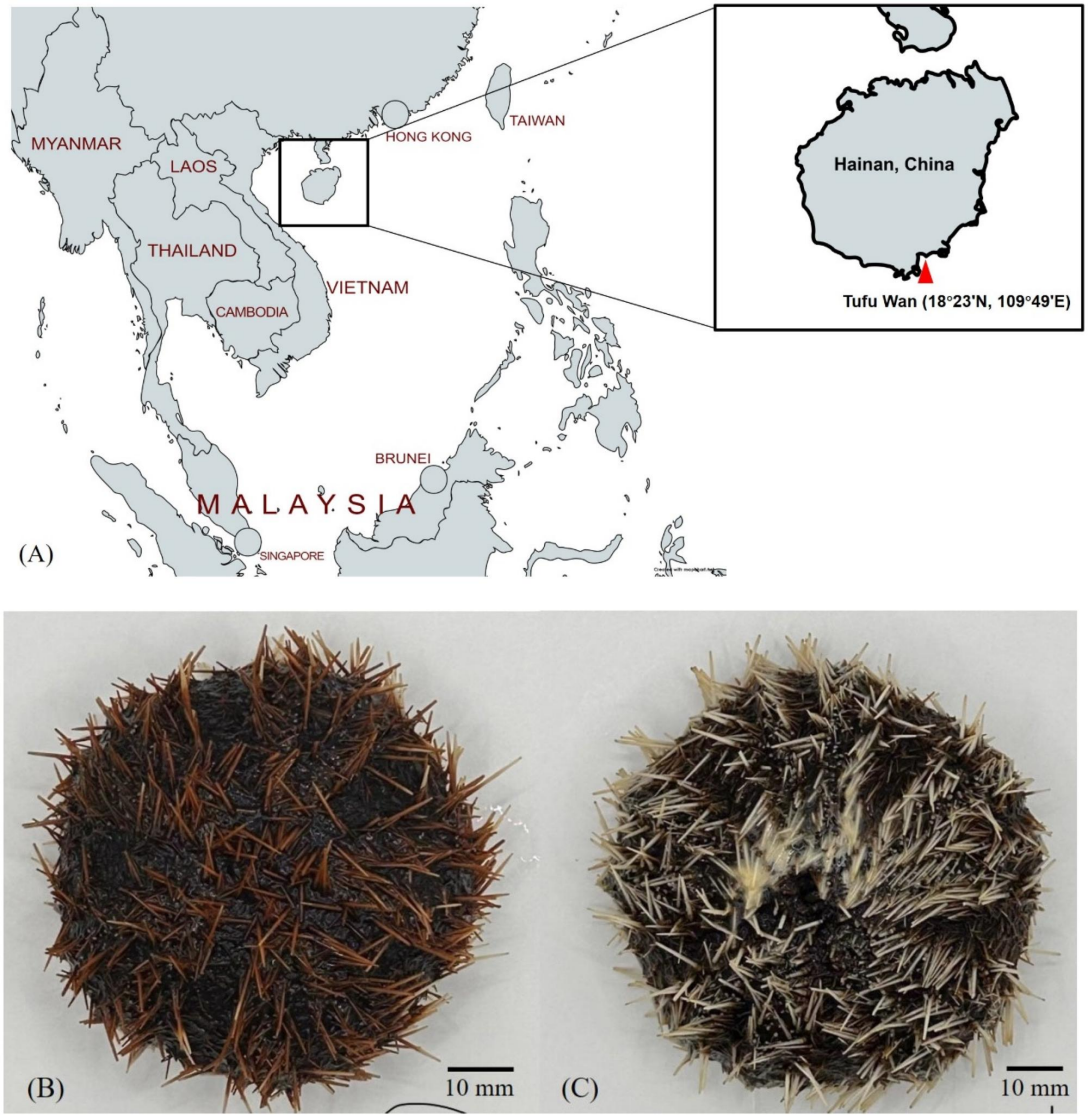
*Tripneustes gratilla* collected from Tufu Wan, Lingshui Li Autonomous County, Hainan Province, China (18°23'N, 109°49'E). the photos of *T. gratilla* with red spines (B) and white spines (C) were captured by the authors at Dalian Ocean University. Scale bar: 10 mm. The DNA samples were stored at -80 °C in Dalian Ocean University and were assigned the numbers DLOU-KLM-SU07 and DLOU-KLM-SU08.

Specimens of *T. gratilla* were collected from Tufu Wan, Lingshui Li Autonomous County, Hainan Province, China (18°23'N, 109°49'E) (Figure 1). The total genomic DNA was extracted from the gonad tissue of each sea urchin using Rapid Animal Genomic DNA Isolation Kit (Sangon Biotech (Shanghai) Co., Ltd., China), following a pre-grind step in liquid nitrogen. DNA samples was sheared into shorter by sonication to mean fragment size 500 bp (Covaris S220), and the fragemented DNA was end-repaired and ligated to sequencing adapters using Hieff NGS® MaxUp II DNA Library Prep Kit for Illumina® (Yeasen Biotechnology (Shanghai) Co., Ltd., China). The DNA libraries were sequenced using the Novaseq 6000 system platform (Illumina, San Diego, USA)/DNBseq-T7 sequencer (BGI, Shenzhen, China) with a 2 × 150 bp paired-end sequence kit, in accordance with the manufacturer’s instructions. The obtained raw reads were subjected to trimming using Fastqc (0.11.2), followed by assembly into contigs using SPAdes (v3.15). The complete mitochondrial genome (mt-genome) was achieved by utilizing the contigs that aligned with the Hawaiian sea urchin (*Tripneustes gratilla*) mitochondrial reference genome (NCBI accession number NC_034770.1) as the seed sequence for the software MITObim (v1.9.1) with 30 iterations. To annotate the protein-coding genes, transfer RNA (tRNA), and ribosomal RNA (rRNA) genes present in the genome, the NCBI Eukaryotic Genome Annotation Pipeline was employed. The DNA samples were stored at −80 °C in the Key Laboratory of Mariculture & Stock Enhancement in North China’s Sea, Ministry of Agriculture and Rural Affairs, Dalian Ocean University, and were assigned the numbers DLOU-KLM-SU07 and DLOU-KLM-SU08 (www.dlou.edu.cn, contact person: Dr. Heng Wang, email: hengwang@dlou.edu.cn).

## Results and discussion

**Figure 2.**
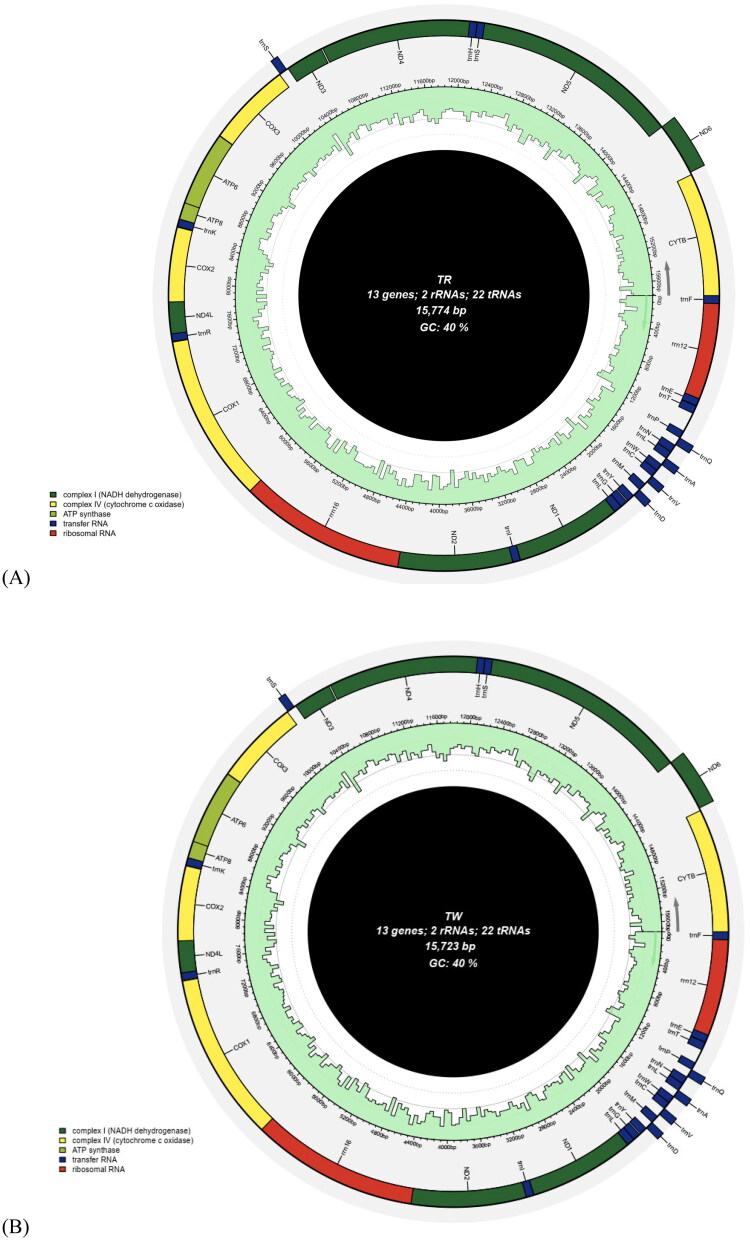
Mitochondrial genome of *Tripneustes gratilla* with red spines (TR) (A) and white spines (TW) (B).

The red-spined *T. gratilla* (TR) mt-genome had a total length of 15,774 bp (NCBI accession number OR227932.1) (Figure 2A). It exhibited a base composition of 16.88% G, 23.35% C, 31.27% A, and 28.50% T. The white-spined *T. gratilla* (TW) mt-genome had a total length of 15,723 bp (accession number OR227933) with a base composition of 16.79% G, 23.40% C, 31.34% A, and 28.47% T (Figure 2B). Both mt-genomes consisted of 13 protein-coding DNA sequences (CDS), 2 rRNA genes, and 22 tRNA genes. The protein-coding DNA sequences encoded various genes, including NADH dehydrogenase subunit 1 to 6 (ND1-6), ND4L, cytochrome b (CYTB), cytochrome c oxidase subunit I to III (COX1-3), ATP synthase F0 subunit 6 (ATP6), and APT8. Most of the mitogenome genes were encoded on the plus strand, except for the ND6 gene and 5 tRNA genes (tRNA-Gln, tRNA-Ala, tRNA-Val, tRNA-Asp, and tRNA-Ser). Most CDS genes initiated with the start codon ATG, while the ND4 gene started with ATC and the ATP8 gene with GTG. The stop codons of the CDS genes were predominantly TAA (9 of 13 genes), while three genes (ND3, ND5, and ND6) terminated with TAG and the CYTB gene had a stop codon of CTA. The size of the 22 tRNA genes ranged from 68 to 73 bp.

**Figure 3.**
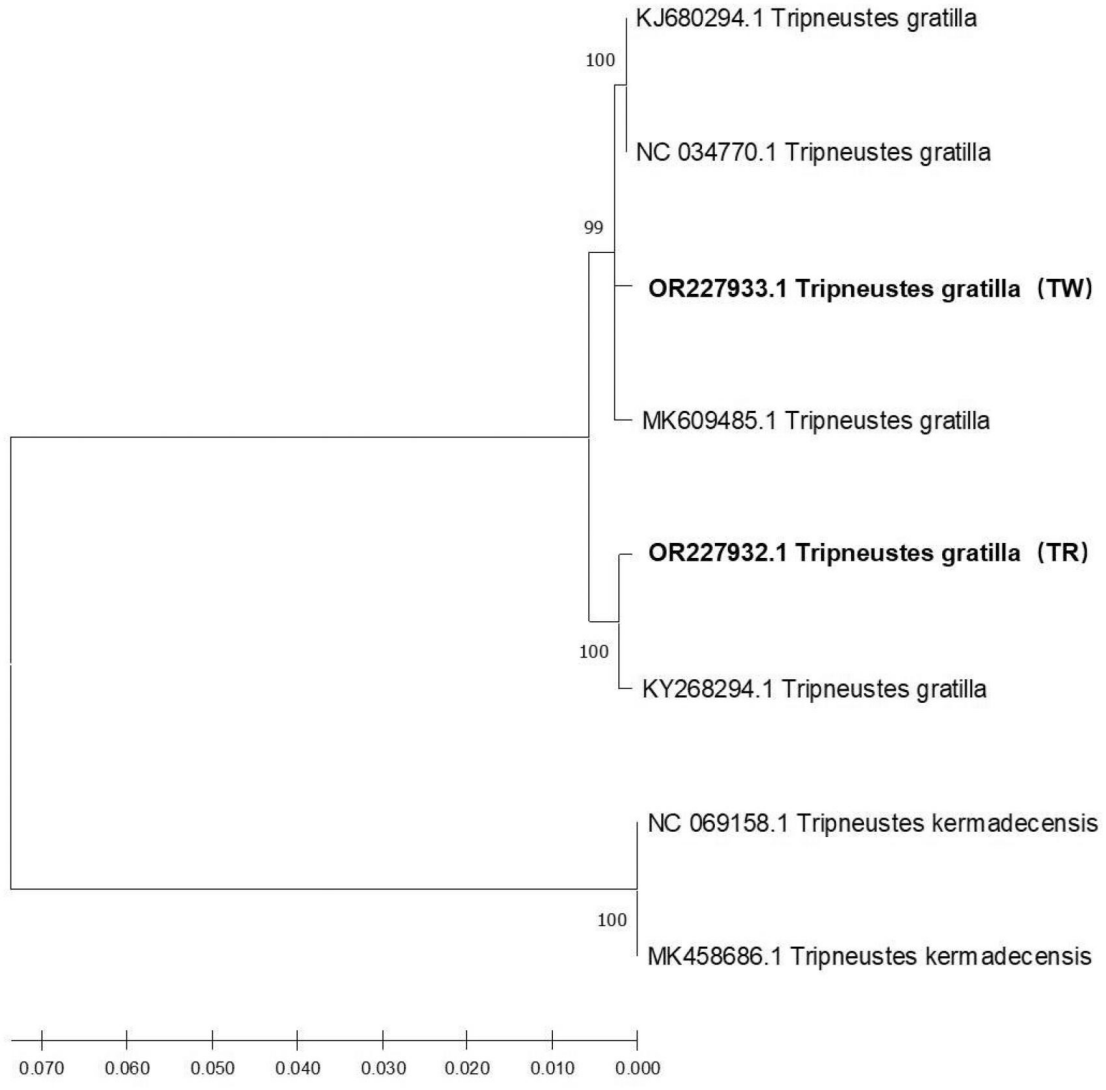
Phylogenetic tree based on eight complete mitochondrial genomic sequences of sea urchins of the genus Tripneustes. The tree was constructed using MEGA X by the Maximum Likelihood method. The numbers above the branches specify bootstrap percentages (1000 replicates). TR represented *T. gratilla* with red spines (NCBI accession number OR227932.1) and TW represented *T. gratilla* with white spines (accession number OR227933.1).

The phylogenetic analysis conducted using the Maximum Likelihood method in MEGA X (Kumar et al. 2018), based on eight complete mitochondrial genomic sequences representing two species of the genus Tripneustes, revealed that the sea urchin samples collected from Tufu Wan, China, grouped together with *T. gratilla* (Figure 3). The comparison of red-spined and white-spined *T. gratilla* sequences showed a high similarity of 98.91%.

Additionally, the red-spined sample exhibited a 99.48% similarity with the mtDNA sequence of *T. gratilla* (accession number KY268294.1) obtained from Oʻahu, Hawai’i (Láruson 2017). Furthermore, the white-spined sample displayed a 99.52% and 99.41% similarity with the sequences of *T. gratilla* (accession numbers KJ680294.1, NC_034770.1 and MK609485.1) collected from Guam and Japan, respectively (Wakayama et al. 2019). However, all the *T. gratilla* showed only 79% similarity with the sequences of *Tripneustes kermadecensis*. Different color-spined species of *T. gratilla* were found in separate branches of the phylogenetic tree of the same sea urchin species. Therefore, further studies involving various locations, morphological observations (Bronstein et al. 2016), age and gender estimations, and analysis of dietary composition are required to develop molecular identification methods for *T. gratilla* with color diversity. These methods could serve as a valuable resource for future investigations into the phylogeny of echinoid species.

## Supplementary Material

Supplemental Material

## Data Availability

The genome sequence data that support the findings of this study are openly available in GenBank of NCBI at [https://www.ncbi.nlm.nih.gov] (https://www.ncbi.nlm.nih.gov/) under the accession no. OR227932.1 (red-spined *T. gratilla*, TR) - OR227933.1 (white-spined *T. gratilla*, TW). The associated BioProject, Sequence Read Archives (SRA), and Bio-Sample numbers are PRJNA1004736, SRR25619644 (TR) - SRR25619647 (TW), and SAMN36950068 (TR) - SAMN36950075 (TW), respectively.
