# Supplementary material for: The complete mitochondrial genome of the tropical sea urchin Tripneustes gratilla (Linnaeus, 1758) with two spine colors
Source: Mitochondrial DNA B Resour. 2024 Jan 3;9(1):15–9. doi: 10.1080/23802359.2023.2298091 (PMC10769132; doi:10.1080/23802359.2023.2298091)
Supplement: Supplemental Material [file TMDN_A_2298091_SM2422.docx]

(A)
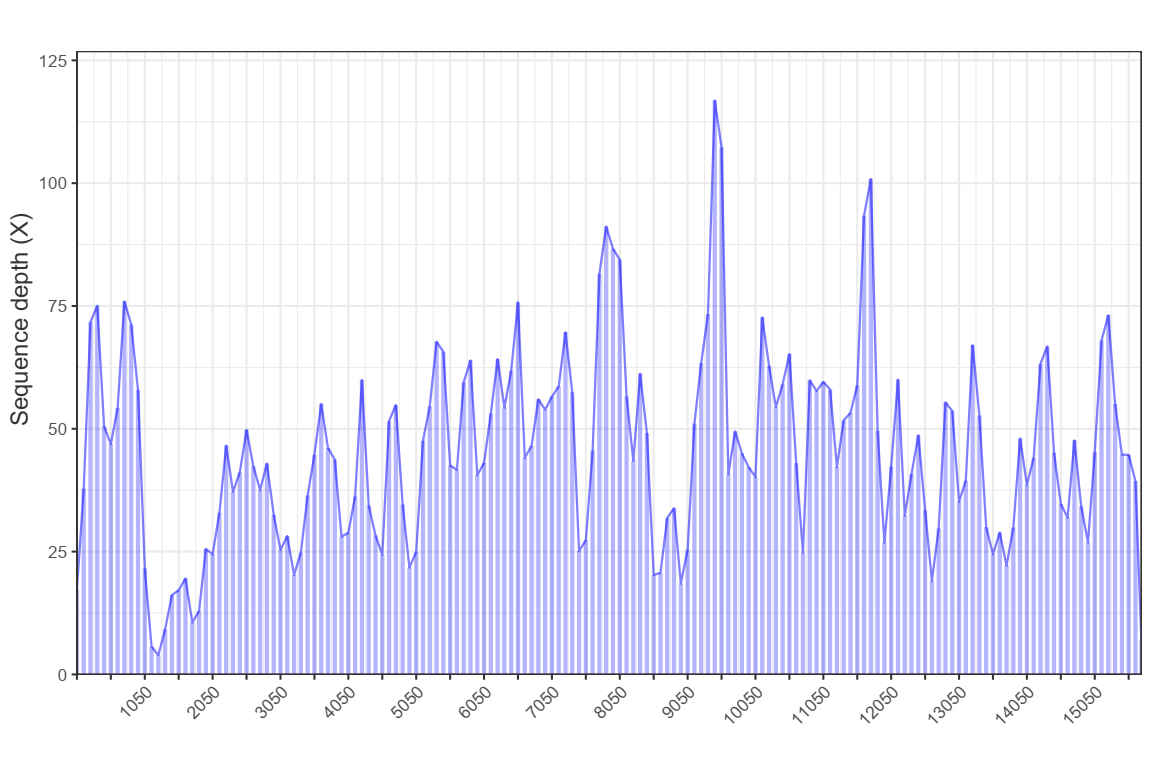
(B)
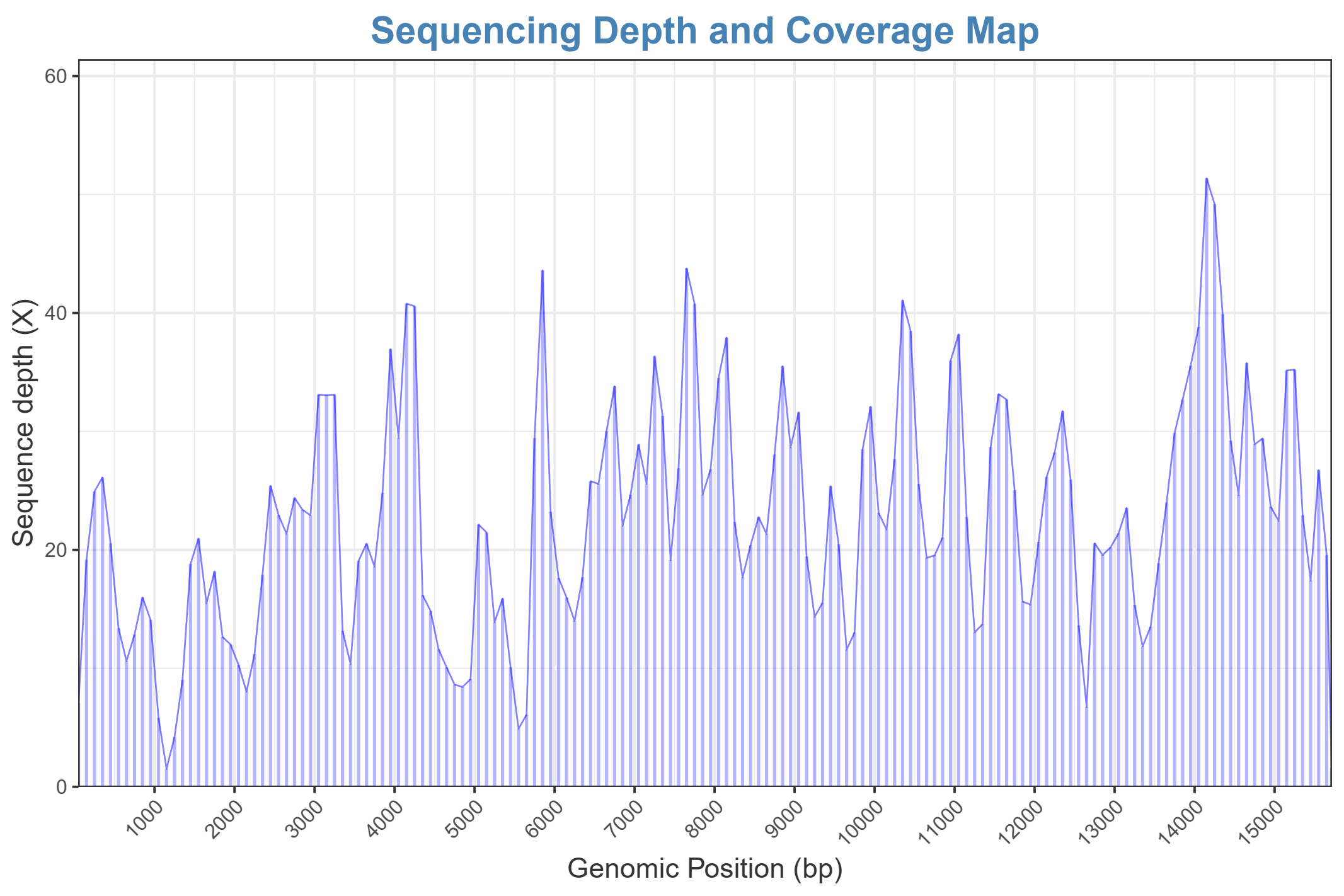


Supplementary Figure 1 Sequencing depth and coverage map for mitochondrial genome of *Tripneustes gratilla* with red spines (A) and white spines (B).
